# Supplementary material for: Identification of a potent V3 glycan site broadly neutralizing antibody targeting an N332gp120 glycan-independent epitope
Source: Nat Immunol. 2026 Feb 3;27(3):572–85. doi: 10.1038/s41590-025-02385-3 (PMC12956603; doi:10.1038/s41590-025-02385-3)
Supplement: Supplementary file 1 [file 41590_2025_2385_MOESM1_ESM.pdf]

nature portfolio

Corresponding author(s): Florian Klein

Last updated by author(s): Sep 2, 2025

# Reporting Summary

Nature Portfolio wishes to improve the reproducibility of the work that we publish. This form provides structure for consistency and transparency in reporting. For further information on Nature Portfolio policies, see our Editorial Policies and the Editorial Policy Checklist.

## Statistics

For all statistical analyses, confirm that the following items are present in the figure legend, table legend, main text, or Methods section.

| n/a | Confirmed | |
|---|---|---|
| ☐ | ☒ | The exact sample size (*n*) for each experimental group/condition, given as a discrete number and unit of measurement |
| ☐ | ☒ | A statement on whether measurements were taken from distinct samples or whether the same sample was measured repeatedly |
| ☐ | ☒ | The statistical test(s) used AND whether they are one- or two-sided<br>*Only common tests should be described solely by name; describe more complex techniques in the Methods section.* |
| ☒ | ☐ | A description of all covariates tested |
| ☒ | ☐ | A description of any assumptions or corrections, such as tests of normality and adjustment for multiple comparisons |
| ☐ | ☒ | A full description of the statistical parameters including central tendency (e.g. means) or other basic estimates (e.g. regression coefficient) AND variation (e.g. standard deviation) or associated estimates of uncertainty (e.g. confidence intervals) |
| ☐ | ☒ | For null hypothesis testing, the test statistic (e.g. *F*, *t*, *r*) with confidence intervals, effect sizes, degrees of freedom and *P* value noted<br>*Give P values as exact values whenever suitable.* |
| ☒ | ☐ | For Bayesian analysis, information on the choice of priors and Markov chain Monte Carlo settings |
| ☒ | ☐ | For hierarchical and complex designs, identification of the appropriate level for tests and full reporting of outcomes |
| ☐ | ☒ | Estimates of effect sizes (e.g. Cohen's *d*, Pearson's *r*), indicating how they were calculated |

*Our web collection on statistics for biologists contains articles on many of the points above.*

## Software and code

Policy information about availability of computer code

| | |
|---|---|
| Data collection | GraphPad Prism V.8: Illustration of data and statistical parameter. Statistical analysis of data.<br>Microsoft Excel V16.16.27 (201012): Illustration of data and statistical parameter. Statistical analysis of data<br>BD FACSDiva 6.1.2: Generation of flow cytometry data.<br>ELISA measurements were performed using Tecan's Sunrise absorbance microplate reader and associated software.<br>Neutralizing activity and luminescence were measures using BertholdTech Tristar2S and associated device software<br>B cell repertoire sequence data was generated by an unbiased template-switch-based NGS approach (Ehrhardt et al., Nature Medicine 2019)<br>Sanger sequencing of antibody v genes were gained by Eurofins genomics.<br>Clinical data for some study individuals was documented in an electronic case report form (eCRF) using the online cohort platform ClinicalSurveys.net hosted by QuestBack, Oslo, Norway on servers of UHC, Cologne, Germany, as part of a software-as-a-service agreement.<br>All computer code and data for the deep mutational scanning is publicly available on GitHub (https://dms-vep.org/HIV_Envelope_TRO11_DMS_007/htmls/all_antibodies_and_cell_entry_overlaid.html). |
| Data analysis | Data, quantifications and statistical analyses were performed using GraphPad Prism V.8, Microsoft Excel V16.16.27 (201012), BD FlowJo V.10, and Geneious Prime (v.2020.2.4). Raw read pre-processing of B cell NGS data was performed with an in-house pipeline primarily basedon self-written Python scripts, IgBLAST, Clustal Omega, and the pRESTO toolkit. Raw IGHV and immunoglobulin light chain variable (IGLV) sequences derived from NGS and single B cell sequencing data were annotated to human V, D, and J germline reference sequences using MiXCR software. All computer code and data for the deep mutational scanning is publicly available on GitHub (https://dms-vep.org/HIV_Envelope_TRO11_DMS_007/htmls/all_antibodies_and_cell_entry_overlaid.html). |

For manuscripts utilizing custom algorithms or software that are central to the research but not yet described in published literature, software must be made available to editors and reviewers. We strongly encourage code deposition in a community repository (e.g. GitHub). See the Nature Portfolio guidelines for submitting code & software for further information.

## Data

Policy information about availability of data

All manuscripts must include a data availability statement. This statement should provide the following information, where applicable:

- Accession codes, unique identifiers, or web links for publicly available datasets
- A description of any restrictions on data availability
- For clinical datasets or third party data, please ensure that the statement adheres to our policy

Nucleotide sequences of bnAb 007 and clonal members have been deposited at GenBank under accession codes XXX. The NGS B cell repertoire data analysed in this study have been deposited in the Sequence Read Archive (SRA) under accession codes SAMN29624595 to SAMN29624713 [https://www.ncbi.nlm.nih.gov/sra?linkname=bioproject_sra_all&from_uid=857338] and the BioProject database under accession code PRJNA857338. Cryo-EM maps and models have been deposited in the Electron Microscopy Data Bank (EMDB) and Protein Data Bank with accession codes: EMD-70018 and PDB ID 9O2Q (007-BG505v2, 0 Fabs bound), EMD-70019 and PDB ID 9O2R (007-BG505v2, 1 Fab bound), EMD-70020 and PDB ID 9O2S (007-BG505v2, 2 Fabs bound), EMD-70021 and PDB ID 9O2T (007-BG505v2, 3 Fabs bound), EMD-70022 and PDB ID 9O2U (007-BG505v2, IgG crosslinked trimer-dimer). Clinical data are available upon request to the corresponding author (F.K.) provided that there is no reasonable risk of deanonymizing study participants and/or may require a Material Transfer Agreement (MTA). Individual donor data cannot be shared due to privacy restrictions. Requests will be responded to within 2 weeks.

## Research involving human participants, their data, or biological material

Policy information about studies with human participants or human data. See also policy information about sex, gender (identity/presentation), and sexual orientation and race, ethnicity and racism.

| | |
|---|---|
| Reporting on sex and gender | In this study, we collected data about the sex of one study individuals. No data was collected on gender. Therefore, we only use the term "sex" in our manuscript. We present data from one female study participants. Samples were collected irrespective of sex or gender. Sex or gender was not relevant to the study design. |
| Reporting on race, ethnicity, or other socially relevant groupings | The origin of samples was Mbeya, Tanzania. |
| Population characteristics | Population characteristic about sex of the described study individuals are as following:<br>1.) Female sex: 100% |
| Recruitment | The donor was recruited from a hospital in Tanzania (Mbeya). Recruitment occurred during routine visits to this facility. Enrollment was unbiased as it was determined by the treating physician without prior selection or self-selection by the patients. However, the physician's choice could introduce potential bias in patient selection. While we recognize this possible source of bias in our recruitment approach, we are confident that it did not influence the study outcomes. |
| Ethics oversight | Large-blood draw samples were collected according to protocols reviewed and approved by the Institutional Review Board (IRB) of the University of Cologne (study protocols 13-364 and 16-054) and local IRBs. Compensation was provided in line with institutional and ethical guidelines to reimburse time and expenses without exerting undue influence. |

Note that full information on the approval of the study protocol must also be provided in the manuscript.

# Field-specific reporting

Please select the one below that is the best fit for your research. If you are not sure, read the appropriate sections before making your selection.

☒ Life sciences ☐ Behavioural & social sciences ☐ Ecological, evolutionary & environmental sciences

For a reference copy of the document with all sections, see nature.com/documents/nr-reporting-summary-flat.pdf

# Life sciences study design

All studies must disclose on these points even when the disclosure is negative.

| | |
|---|---|
| Sample size | No formal sample size calculation was performed. The study aimed to identify potent broadly neutralizing antibodies from a single HIV-1 elite neutralizer by analyzing all available HIV-1–reactive B cell clones isolated from this donor. Sample size was therefore determined by biological availability rather than statistical power considerations. The total number of clones analyzed was sufficient to comprehensively investigate all isolated HIV-1-reactive B cell clones. |
| Data exclusions | For NGS data, reads were initially filtered for a mean Phred score of 25 and read-lengths of at least 250 bp. Consensus sequences (based on UMIs) were excluded, when the corresponding UMI was found less than 3 times. For sequence analysis of single cell-derived v gene sequences, chromatograms were first filtered based on a minimum Phred score of 28 and a length of at least 240 nucleotides. Sequences were then processed using IgBLAST to annotate them, and trimmed to extract only the variable region, extending from FWR1 to the end of the J gene. Base calls within the variable region with a Phred score below 16 were masked. Sequences containing over 15 masked nucleotides, stop codons, or frameshift mutations were excluded from subsequent analysis. No other data were excluded from analyses.<br><br>All anti-HIV-1 bNAbs reference antibodies were functionally validated through neutralization assays against the global HIV-1 pseudovirus |

panel. The resulting $IC_{50}$ and $IC_{80}$ values for each antibody were compared to historical data available in the CATNAP database. Only those antibodies exhibiting less than a threefold deviation in $IC_{50}/IC_{80}$ values relative to the reference data were included in subsequent functional analyses.

| | |
|---|---|
| Replication | For antibody screening assays, the neutralizing activity of monoclonal antibodies was first assessed without duplicates. Determination of the neutralizing activity of antibodies against other virus panels were conducted in duplicates and the geometric mean was reported. All attempts at replication were successful. |
| Randomization | Randomization was relevant for the in vivo treatment experiments; mice were randomized into treatment or control groups based on viral load, age, and time since humanization to ensure even distribution across groups. |
| Blinding | Researches conducting experiments were aware of the study ID for each individual they handled, but they were blinded to the participants' clinical data. The aim was to identify and characterize a broadly neutralizing antibody (bNAb) from a single HIV-1 elite neutralizer. All experiments were exploratory and descriptive in nature. Data collection and analysis involved quantitative, assay-based measurements (e.g., neutralization assays, binding analyses, structural studies) that were not influenced by investigator bias. Therefore, blinding was not applicable to the experimental design. |

# Reporting for specific materials, systems and methods

We require information from authors about some types of materials, experimental systems and methods used in many studies. Here, indicate whether each material, system or method listed is relevant to your study. If you are not sure if a list item applies to your research, read the appropriate section before selecting a response.

## Materials & experimental systems

| n/a | Involved in the study |
|---|---|
| ☐ | ☒ Antibodies |
| ☐ | ☒ Eukaryotic cell lines |
| ☒ | ☐ Palaeontology and archaeology |
| ☐ | ☒ Animals and other organisms |
| ☐ | ☒ Clinical data |
| ☒ | ☐ Dual use research of concern |
| ☒ | ☐ Plants |

## Methods

| n/a | Involved in the study |
|---|---|
| ☒ | ☐ ChIP-seq |
| ☐ | ☒ Flow cytometry |
| ☒ | ☐ MRI-based neuroimaging |

## Antibodies

| | |
|---|---|
| Antibodies used | Anti-human CD20-AF700 (clone 2H7), BD Bioscience, Cat#560631, RRID: AB_2687799, dilution 1:80<br>Anti-human CD19-AF700 (clone HIB19), BD Bioscience, Cat#557921, RRID: AB_396942, dilution 1:80<br>Anti-human IgG-APC (clone G18-145), BD Bioscience, Cat#550931, RRID AB_2738854, dilution 1:20<br>Anti-human CD27-PE (Clone M-T271), BD Bioscience, CAT#560985, RRID: AB_10563213, dilution 1:20<br>Peroxidase AffiniPure Goat Anti-human IgG, Fcγ fragment specific, Jackson ImmunoResearch, Cat#109-035-098, RRID: AB_237586<br>IgG1 kappa from human myeloma plasma (Sigma Aldrich, cat. no. I5154), dilution 1:5000<br>Monoclonal anti-HIV-1 bNAb 10-1074, sequence retrieved from CATNAP under record #2777, starting concentration of neutralization assays of 10, 25 or 50 µg/ml<br>Monoclonal anti-HIV-1 bNAb BG18, sequence retrieved from CATNAP under record #3623, starting concentration of neutralization assays of 10, 25 or 50 µg/ml<br>Monoclonal anti-HIV-1 bNAb PGT121, Genbank ID heavy chain: JN201894, light chain: JN201911, starting concentration of neutralization assays of 10, 25 or 50 µg/ml<br>Monoclonal anti-HIV-1 bNAb PGT128, Genbank ID heavy chain: JN201900, light chain: JN201917, starting concentration of neutralization assays of 10, 25 or 50 µg/ml |
| Validation | All antibodies from BD bioscience and Jackson ImmunoResearch were controlled for human reactivity during manufcaturer's quality control. All anti-HIV-1 bNAbs were tested against the global pseudovirus panel to guarantee stability and functionality. All anti-HIV-1 bNAbs reference antibodies were functionally validated through neutralization assays against the global HIV-1 pseudovirus panel. The resulting $IC_{50}$ and $IC_{80}$ values for each antibody were compared to historical data available in the CATNAP database. Only those antibodies exhibiting less than a threefold deviation in $IC_{50}/IC_{80}$ values relative to the reference data were included in subsequent functional analyses. |

## Eukaryotic cell lines

Policy information about cell lines and Sex and Gender in Research

| | |
|---|---|
| Cell line source(s) | TZM-bl cells (NIH AIDS Reagent Program, Ref# ARP5011), HEK293T (American Type Culture Collection, Ref# CRL-3216), HEK293-6E cells (National Research Council of Canada, Cat. No. NR16-179/2017E). The sex of all cells was female. |

| | |
|---|---|
| Authentication | Cell line were authenticated with STR analysis. |
| Mycoplasma contamination | Cell line was tested negative for mycoplasma contamination. |
| Commonly misidentified lines (See ICLAC register) | No commonly misidentified cell lines were used in the study. |

## Animals and other research organisms

Policy information about studies involving animals; ARRIVE guidelines recommended for reporting animal research, and Sex and Gender in Research

| | |
|---|---|
| Laboratory animals | NOD.Cg-Rag1tm1momIl2rgtm1Wjl/SzJ (NRG) mice were obtained from The Jackson Laboratory<br>B6.Cg-Fcgrttm1Dcr Prkdcscid Tg(FCGRT)32Dcr/DcrJ (FcRn) mice were obtained from The Jackson Laboratory |
| Wild animals | No wild animals were used for this study |
| Reporting on sex | For treatment experiments using NOD.Cg-Rag1tm1momIl2rgtm1Wjl/SzJ (NRG) mice obtained from The Jackson Laboratory:<br>14 male mice, 19 female mice<br><br>For determination of antibody PKs in vivo using B6.Cg-Fcgrttm1Dcr Prkdcscid Tg(FCGRT)32Dcr/DcrJ (FcRn) mice obtained from The Jackson Laboratory:<br>12 female mice (007, 10-1074, 3BNC117) |
| Field-collected samples | No field samples were collected in this study. |
| Ethics oversight | All procedures involving mice in this study were sanctioned by the State Agency for Nature, Environmental Protection, and Consumer Protection North Rhine-Westphalia (LANUV). |

Note that full information on the approval of the study protocol must also be provided in the manuscript.

## Clinical data

Policy information about clinical studies
All manuscripts should comply with the ICMJE guidelines for publication of clinical research and a completed CONSORT checklist must be included with all submissions.

| | |
|---|---|
| Clinical trial registration | Studies have not been registered |
| Study protocol | Study protocols will be made available on request from the corresponding author. |
| Data collection | Data collection was carried out at each site using either local data monitoring systems or the centralized online cohort platform, ClinicalSurveys.net. The collected data were then harmonized and consolidated by staff at the University Hospital Cologne. |
| Outcomes | Non-interventional study. No outcome parameters were defined. |

## Plants

| | |
|---|---|
| Seed stocks | No plants were used in this study |
| Novel plant genotypes | No plants were used in this study |
| Authentication | No plants were used in this study |

## Flow Cytometry

### Plots

Confirm that:

☒ The axis labels state the marker and fluorochrome used (e.g. CD4-FITC).

☐ The axis scales are clearly visible. Include numbers along axes only for bottom left plot of group (a 'group' is an analysis of identical markers).

☒ All plots are contour plots with outliers or pseudocolor plots.

☒ A numerical value for number of cells or percentage (with statistics) is provided.

### Methodology

| | |
|---|---|
| Sample preparation | PBMCs of study individuals were purified by standard density gradient centrifugation using Histopaque (Sigma Aldrich) and LeucoSep tubes (Greiner Bio-one). Cells were sorted at -150°C in 90% (v/v) FBS (Sigma Aldrich) and 10% (v/v) DMSO (Sigma Aldrich). For single B cell sorts, cells were stained with anti-human CD19-AF700 (BD Bioscience), anti-human IgG-APC (BD Bioscience), DAPI (Thermo Fisher), and the HIV-1 env bait BG505.SOSIP.664-GFP for 30 min on ice. Env-reactive CD19+IgG +DAPI- single cells were sorted into 96-well plates containing 4 µl of lysis buffer. |
| Instrument | FACSAria Fusion (Becton Dickinson) |
| Software | BD FACSDIVA, FlowJo10 |
| Cell population abundance | Post-sort fractions were not re-analyzed. |
| Gating strategy | Gating on lymphocytes, live cells, CD19+ and IgG+ and the GFP+ (BG505.SOSIP.664-GFP) for HIV-1 reactive cells. |

☒ Tick this box to confirm that a figure exemplifying the gating strategy is provided in the Supplementary Information.
