# Supplementary material for: Identification of a potent V3 glycan site broadly neutralizing antibody targeting an N332gp120 glycan-independent epitope
Source: Nat Immunol. 2026 Feb 3;27(3):572–85. doi: 10.1038/s41590-025-02385-3 (PMC12956603; doi:10.1038/s41590-025-02385-3)

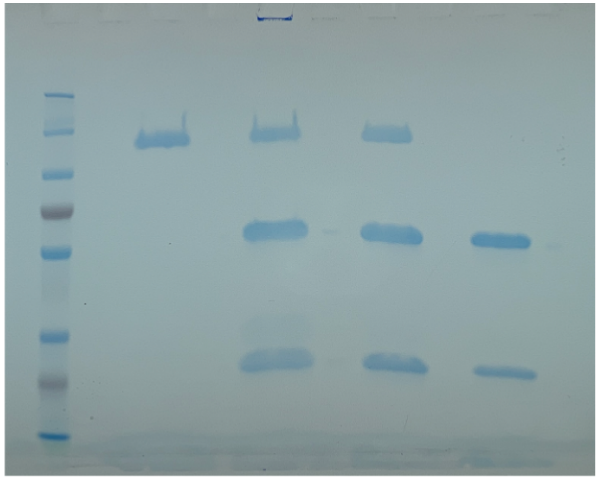
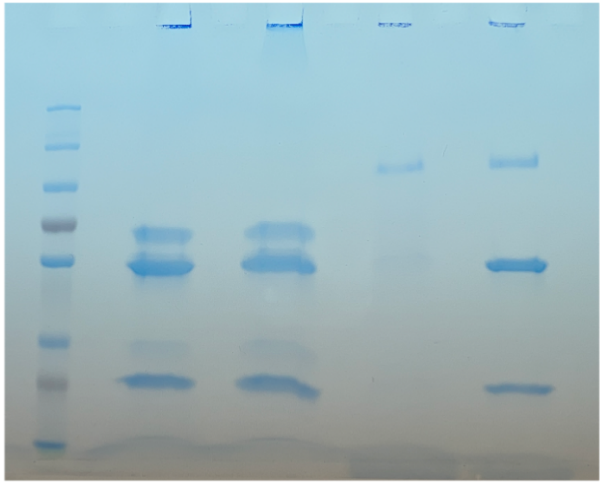
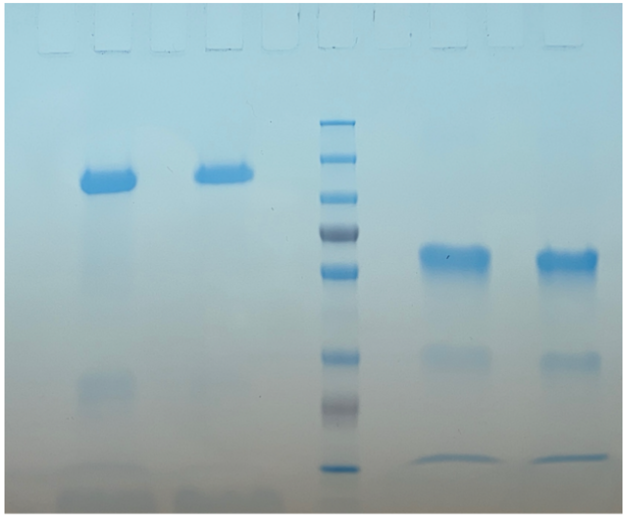

Supplement: Supplementary file 3 [file 41590_2025_2385_MOESM4_ESM.pdf]
